# Temperature management for patients without brain injury in Australia and New Zealand ICUs: a point prevalence study

**DOI:** 10.1186/cc10665

**Published:** 2012-03-20

**Authors:** NE Hammond, M Saxena, P Young, C Taylor, I Seppelt, P Glass, J Myburgh

**Affiliations:** 1 George Institute for Global Health, Sydney, Australia

## Introduction

Our primary aim was to determine the frequency of use of pharmacological and physical cooling strategies in ICU patients in current Australian and New Zealand (ANZ) practice. These patients had sepsis and inflammation but did not have neurological injury or recent surgery. We also aimed to establish current indications for use of antipyretics in these patients, as well as information on the prevalence of fever and the methods to measure temperature.

## Methods

This point prevalence study was conducted on 17 November and 15 December 2010 in 38 ICUs in ANZ. We identified a cohort of patients with sepsis and inflammation without neurological injury or recent surgery.

## Results

**Figure 1:**
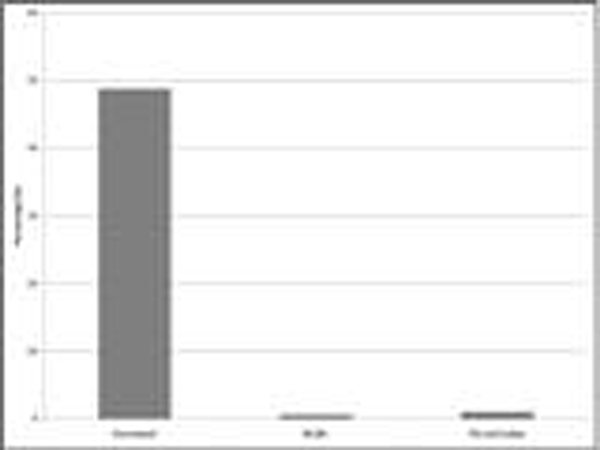
**Type of antipyretic and physical cooling used on the study day (*n *= 311)**.

**Figure 2:**
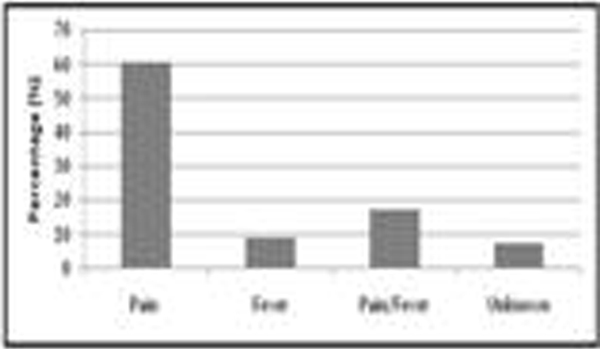
**Indication for paracetamol administration (*n *= 152)**.

Of 506 patients surveyed on the point prevalence days, 311 were identified to have sepsis in the absence of neurological injury or recent surgery. These patients had peak temperature of 37.3°C (SD 0.8°C). In 32.2% (*n *= 100/311) the peak temperature was above 38°C. Paracetamol was used in 152/311 (48.8%), nonsteroidal anti-inflammatory drugs (NSAIDS) in 2/311 (0.6%) and physical cooling in 3/311 (1.0%) (Figure 1). Paracetamol was administered for pain in 92/152 (60.5%) for both pain and fever in 26/152 (17.1%); and for fever alone in 14/152 (10%) (Figure 2). For the 40 patients who received paracetamol for an indication of fever, the peak recorded temperature was 38.3°C (SD 0.8°C). The peak temperature for patients receiving physical cooling was 39.2°C (SD 0.9°C). Temperature measurement were mainly noncore (*n *= 251/311) with axillary (37%; *n *= 116/311) and tympanic (35%; *n *= 110/311) the most common sites.

## Conclusion

Pharmacological antipyretics are used regularly for pain management rather than fever management, with paracetamol the most common antipyretic therapy. The use of NSAIDS and physical cooling was rare. Noncore temperature measurements were common.

